# Bias and Sensitivity in the Placement of Fossil Taxa Resulting from Interpretations of Missing Data

**DOI:** 10.1093/sysbio/syu093

**Published:** 2014-11-27

**Authors:** Robert S. Sansom

**Affiliations:** Faculty of Life Sciences, University of Manchester, Manchester M13 9PT, UK

**Keywords:** Missing data, paleontology, phylogeny, soft-bodied, stem-group, taphonomy

## Abstract

The utility of fossils in evolutionary contexts is dependent on their accurate placement in phylogenetic frameworks, yet intrinsic and widespread missing data make this problematic. The complex taphonomic processes occurring during fossilization can make it difficult to distinguish absence from non-preservation, especially in the case of exceptionally preserved soft-tissue fossils: is a particular morphological character (e.g., appendage, tentacle, or nerve) missing from a fossil because it was never there (phylogenetic absence), or just happened to not be preserved (taphonomic loss)? Missing data have not been tested in the context of interpretation of non-present anatomy nor in the context of directional shifts and biases in affinity. Here, complete taxa, both simulated and empirical, are subjected to data loss through the replacement of present entries (1s) with either missing (?s) or absent (0s) entries. Both cause taxa to drift down trees, from their original position, toward the root. Absolute thresholds at which downshift is significant are extremely low for introduced absences (two entries replaced, 6% of present characters). The opposite threshold in empirical fossil taxa is also found to be low; two absent entries replaced with presences causes fossil taxa to drift up trees. As such, only a few instances of non-preserved characters interpreted as absences will cause fossil organisms to be erroneously interpreted as more primitive than they were in life. This observed sensitivity to coding non-present morphology presents a problem for all evolutionary studies that attempt to use fossils to reconstruct rates of evolution or unlock sequences of morphological change. Stem-ward slippage, whereby fossilization processes cause organisms to appear artificially primitive, appears to be a ubiquitous and problematic phenomenon inherent to missing data, even when no decay biases exist. Absent characters therefore require explicit justification and taphonomic frameworks to support their interpretation.

The loss of anatomical features through decay is a pervasive and inescapable factor in the preservation of fossils. As a logical consequence of this incompleteness, it is necessary to ask: is any particular absent anatomical feature, such as an appendage, tentacle, or nerve cord, missing from a fossil because it was never there in the original, living organism, or instead because it was lost at some point between death and observation? Making the distinction between the phylogenetic absence of synapomorphies and their taphonomic loss (i.e., non-fossilization) is crucial if we hope to reconstruct the morphology and evolutionary significance of fossil taxa; it is the very combination of absent and present morphological characters that is fundamental to interpretation of the phylogenetic affinity of a fossil and placement on a stem-lineage. Only by placing an extinct organism in a phylogenetic framework can we hope to unlock the sequence of character changes taking place along a stem-lineage, and thus the nature of evolutionary origins and transitions. Furthermore, without knowledge of fossil affinities, it would not be possible to calibrate molecular clocks to provide accurate timescales for evolutionary events (Donoghue and Benton 2007). It is therefore crucial that the complement of morphological characters present and absent in extinct organisms is explicitly understood so that fossil phylogeny can be elucidated robustly.

Morphological data require logical consideration with respect to rationale of character coding which can introduce effectively missing data through inapplicable entries (Strong and Lipscomb 1999; Forey and Kitching 2000; Brazeau 2011). Fossil taxa, however, are afflicted by a different and more problematic source of missing data—incomplete preservation. The role of missing morphology in phylogeny reconstruction, irrespective of source, has been disputed (Scotland et al. 2003; Wiens 2004). Analyses of simulated and empirical taxa are in general agreement that incomplete taxa and characters need not represent a barrier to accurate reconstruction of relationships (Huelsenbeck 1991; Kearney and Clark 2003; Wiens 1998, 2003a, 2003b, 2006; Prevosti and Chemisquy 2010; Wiens and Morrill 2011). What remains unclear is how different interpretations of non-present morphology in fossils affect phylogenetic inference—missing data have been treated as just that in analyses, missing, without accounting for the possibility that missing fossil morphology is conflated with absence. Furthermore, analyses of missing data have generally focused on *accuracy* of phylogenetic inference, the implicit assumption being that missing data or errors cause no systematic changes in relationships. There is, however, strong reason to suspect that both factors—inability to distinguish absence from non-preservation and directional shifts in phylogenetic affinity—present a problem for fossil taxa.

With respect to directional shifts, removal of soft-tissue characters from morphological phylogenies of extant taxa (i.e., simulation of data loss during fossilization) has revealed that missing data cause not only inaccuracy of phylogenetic inference, but also systematic errors; missing soft data caused taxa to be placed lower in trees, closer to the root of phylogenies, than random missing data in the same proportion (Sansom and Wills 2013). This systematic shift has ramifications for the evolutionary significance that we apply to fossil taxa, which in the most part are missing soft-tissue morphology. Furthermore, taphonomic experiments on soft-bodied forms have also revealed directional shifts. Patterns of taphonomic character loss observed during decay of chordates reveal systematic loss of synapomorphies, and thus the morphology of decayed bodies appears artifactually primitive (Sansom et al. 2010a, 2011, 2013). This bias of stem-ward slippage results from an unexpected correlation between the order of loss of characters in decay sequences and their apomorphic rank (synapomorphies are lost before plesiomorpies); decayed crown-group fossils appear as members of the stem-group following incomplete preservation of characters. Both these studies reveal that incomplete morphology resulting from fossilization causes biases whereby the phylogenetic affinity of a taxon is systematically shifted from its original position to an erroneous position lower in phylogenies. As such, the previous focus on phylogenetic accuracy with respect to missing data means that the systematic shifts have not been characterized, either in terms of direction, sensitivity, or prevalence.

The potential role of directional shifts is especially important in paleontological cases due to the difficulties in distinguishing phylogenetic absence from taphonomic loss. Donoghue and Purnell (2009) have demonstrated the pivotal role this can play in interpretations of fossil affinity, and how easily errors can occur. For example, the Devonian taxon *Scaumenella*, with its absence of complex features, was initially interpreted as a primitive chordate, yet subsequent comparative taphonomy revealed this absence to be a result of incompleteness; instead these fossils are the rotted remains of the acanthodian *Triazeugacanthus*, and therefore a boney jawed vertebrate (Beland and Arsenault 1985; Donoghue and Purnell 2009). Likewise, the discovery of well-preserved specimens of the Cambrian arthropod *Kunmingella* has revealed segments and appendages previously envisaged as not present (Hou et al. 1996, 2010; Shu et al. 1999). Revising this absence to presence subsequently changed interpretations of the phylogenetic affinity of this stem-crustacean taxon, as well as that of related taxa (Hou et al. 2010).

Due to pervasive incompleteness, the problem of taphonomic loss versus phylogenetic absence is relevant when coding any fossil taxon. This includes organisms with hard skeletons but it is especially problematic when considering soft-bodied organisms, that is, those that lack readily fossilizable biomineralized tissues. Soft organisms make up a large part of the tree of life—25 of 33 putative metazoan phyla, or about 90% of Phanerozoic species that have existed (Paul 1998). Furthermore, the deep branches of the tree of life subtending the origin and diversification of phyla are composed of entirely soft-bodied organisms, including those clades that do biomineralize (Murdoch and Donoghue 2011). Where the geological circumstances are such that preservation of soft bodies occurs (Konservat-Lagerstätten), the resulting fossils provide unique evolutionary insight into important evolutionary events. A prominent example is the Cambrian explosion of animal phyla as revealed at the Burgess Shale from the Cambrian of Canada. Here, and in similar deposits from around the world, soft-bodied organisms were rapidly buried in fine sediments with restricted oxidant flow which resulted in thin carbonaceous films left by the soft bodies of arthropods, worms, molluscs, and even chordates (Briggs et al. 1994; Hou et al. 2004). Similarly valuable insights are also provided by other Konservat-Lagestätte including the Devonian Hunsrück Slates (Bartels et al. 2009), Carboniferous Mazon Creek concretions (Shabica and Hay 1997), and Cretaceous Jehol Biota (Zhou et al. 2003). The morphology of even the best preserved soft-bodied fossils is, however, subject to complex processes of change and incompleteness resulting from decay. Under these circumstances, the distinction between phylogenetic absence and taphonomic loss of fossil morphology is even harder to make, and these interpretations of non-preserved characters can be, and will be, frequently conflated (Donoghue and Purnell 2009; Sansom et al. 2011). Interpretation of non-present characters is difficult in this context. Reference to better preserved specimens or specimens of close relatives can inform interpretations of non-present characters as missing (?), but interpretations of non-present characters as absent (0) is more problematic. In either case, justifications are rarely explicit. Unless properly taken into account and corrected for, taphonomic processes undermine our ability to identify the affinity of extinct organisms, both soft and hard.

In this study, the role of taphonomic loss of data on phylogenetic inference is investigated using both simulated taxa and empirical taxa. Phylogenetic analysis of fossil organisms treats morphological characters as discrete observations, often presence and absence (0 vs. 1), which are used to construct the shortest possible tree. Studies of fossil morphology almost always use parsimony algorithms, universally so in the case of soft-bodied fossils. Large amounts of anatomy will be inescapably missing, even with fine preservation. Any anatomical feature or character not observed in a fossil could be interpreted as missing, and coded accordingly as ?; this leaves open the possibility that the structure was present in the original organism but was not preserved. It also adds no further information for our elucidation of relationships. Alternatively, any non-present anatomical feature could be interpreted as absent, most often coded as 0. In the experiments conducted here, the impact of taphonomic loss of data is scrutinized by introducing either missing data (?) or absent entries (0) to taxa for which data already exist, and then quantifying alterations to the phylogenetic position of those taxa in terms of directional shifts. The null expectation for introduced absent entries (replacing 1s with 0s) is that taxa will move from their original position to a position closer to the root of the tree as they become more similar to the outgroup condition (assumed to be plesiomorphic and logically mostly 0s). The more pertinent question of interest concerns the threshold values at which introduced absences cause significant shift of taxa from its original position. How many times does non-preservation of anatomical features have to be conflated with phylogenetic absence to cause stem-ward slippage and problems for reconstruction of evolutionary relationships? These thresholds are interrogated in terms of both absolute number of errors and percentage errors in both simulated and empirical data sets. Conversely, the effects of overly cautious coding of non-present characters and the sensitivity of empirical fossil taxa to upward shift are investigated. To ground truth these findings, the same rationale was applied to empirical fossil taxa that could have been subjected to conflation of non-preservation and absence to identify the thresholds at which they shift significantly up. Empirical fossil taxa serve as real-world examples where directional shifts could have already affected their position.

## Methods

### Rationale

It is the position of an extinct taxon in a phylogeny relative to extant taxa and relative to the root that is of relevance to evolutionary inference, be that reconstruction of character evolution or estimation of evolutionary rates and timescales. The position of a taxon is quantified here by calculating the distance of a taxon from the root of a tree, averaged for all fully resolved, most parsimonious trees (Sansom and Wills 2013). When individual taxa are manipulated in any way (in this instance modeling fossilization through introduction of missing entries or absent entries), their position following subsequent searches could either remain the same, or be different; in the case of change, a taxon might move down the tree, toward the root, or up the tree away from the root and its original position, each having different evolutionary implications (Sansom and Wills 2013). It is these kinds of perturbations that are used to investigate the role of fossilization in phylogenetic inference. A lateral shift of a taxon does not affect the distance from the root and accordingly does not affect interpretation of a taxon as fundamentally primitive or derived; lateral shifts are therefore treated as neutral here. The null expectation for the introduction of random missing data (replacing 0 or 1 entries with ? entries) is that greater amounts will introduce more noise, but that any changes will be random and have no systematic direction. There are sound theoretical and empirical reasons to expect neutral changes (Wiens 1998, 2003a, 2003b, 2006), but they have not been tested in terms of directional shifts. Furthermore, it is necessary to perform these tests as the resulting distributions serve as a control for testing the effects of introduction of random absences.

### Data

Simulated data sets are an important tool for investigating questions about phylogenetic accuracy because we have a starting point for a ‘true’ phylogeny and we understand the parameters underlying their creation (Wiens 1998). Furthermore, there is a long history of utility of simulated data sets in the context of missing data (Huelsenbeck 1991, Wiens 2004, 2003a, 2003b). Random branch lengths were assigned to random trees, and those trees were used to generate Neyman/Jukes-Cantor data according to the *xread* command of TNT (Goloboff et al. 2008). The script used to simulate starting trees and data sets is provided in the Supplementary Information (available from http://www.sysbio.oxfordjournals.org/; Dryad http://dx.doi.org/10.5061/dryad.7tq20, end script). The resulting data sets consisted of 20 taxa (19 ingroup and 1 outgroup of all 0s) with 100 informative and binary characters (all uninformative characters were removed). These dimensions (20×100) are suitable to investigate the movement of taxa within trees and roughly approximate to the size of morphological data sets of soft-bodied organisms (see below) as well as data sets used in previous missing data simulations (Wiens 1998, 2003a, 2003b). Given random tree generation, tree shapes ranged from balanced to ladderized.

Five published morphological data sets of extant, non-biomineralized, soft-bodied clades are used as a point of comparison and to ground-truth tests: Scalidophora (principally Priapulida, Wills et al. 2012), Arthropoda (Legg et al. 2012), Cephalopoda (Lindgren et al. 2004), Hemichordata (principally enteropneusts, Cameron 2005), and Annelida (Zrzavý et al. 2009). The empirical data matrices were edited for the purpose of the analyses presented here. Only extant taxa are the subject of the introduction of missing data and absences because they have not been previously afflicted by taphonomic processes. Extinct taxa were therefore removed for these data sets (i.e., Legg et al. 2012; Wills et al. 2012). Uninformative characters were also removed. Some of the empirical data sets had very little resolution in the strict consensus tree (Lindgren et al. 2004; Zrzavý et al. 2009); removal of selected wildcard taxa improved resolution and provided a starting point for phylogeny manipulation in these cases. The dimensions of the data sets are given in Table 1. To analyze the phylogenetic properties of fossil taxa, versions of the matrices of Legg et al. (2012) and Wills et al. (2012) including fossil taxa (102 and 26, respectively) were used, alongside matrices of Daley et al. (2009) and Ma et al. (2013; 15 of 17 and 24 of 27 taxa extinct, respectively).

**Table 1.** Median thresholds for significant taxon shift by data set

Downward movement				Introduced absences (1s to 0s)			Introduced missing (1s to ?s)		
	Data set dimensions				Median replacements			Median replacements	
Data set, extant	Characters	Taxa	Taxa for thresholds	% Reaching thresholds	No. of entries	Percent (%)	% Reaching Thresholds	No. of entries	Percent (%)
Simulated (×40)	100	20	733	99.7	2	6	85.3	10	50
Cameron (2005)	70	20	19	100	1	4	84.2	9	51
Wills et al. (2012)	71	23	21	100	1	3	100	2	10
Legg et al. (2012)	427	69	66	100	13	21	31.8	13	21
Lindgren et al. (2004)	71	34	31	96.7	2	10	77.4	1	5
Zrzavý et al. (2009)	83	64	62	100	2	22	72.6	2	18
Total empirical			199	99.5	2	11	63.2	3	13
							Including non-threshold	14	51

Upward movement				Introduced presences (0s to 1s)		
	Data set dimensions				Median replacements	
Data set, fossil	Characters	Taxa	Taxa for thresholds	% Reaching thresholds	No. of entries	Percent (%)
Wills et al. (2012)	78	49	26	84.6	2	10
Legg et al. (2012)	554	173	96	96.9	55	50
Daley et al. (2009)	38	17	13	92.3	2	17
Ma et al. (2013)	38	27	22	100	2	11
Total empirical with Legg				94.9	22	25
Total empirical without Legg				91.8	2	11

### Introduction of Random Missing Data and Absences

As a benchmark for the original position of a taxon in the starting tree, the distance of a taxon from the root, in terms of the number of intervening nodes, is averaged for all most parsimonious trees. Trees are found using traditional searches in TNT (*mult*) with 100 random addition sequences holding 1000 trees maximum per replication, 10,000 maximum overall. All trees are fully resolved; loss of resolution or branch collapsing does not, therefore, affect calculations of taxon distance from root, in itself. Furthermore, because taxon height is interrogated and averaged for all most parsimonious trees, downward taxon shifts do not result from branch collapse as they would for consensus trees. To evaluate the role of random missing data on the position of taxa in phylogenies, different proportions of missing entries were introduced to individual taxa: 1%, 2%, 5%, 10%, 20%, 50%, 80%, and 95%. At each level of missing data, entries of the original matrix are replaced randomly for 100 random iterations in one of two different ways: 0s and 1s replaced with ? (random missing data), or 1s replaced with 0s (taphonomic loss interpreted as absence). For the latter, only characters that are present (1s) in any particular taxon are selected for random replacement thus avoiding replacement of 0s with 0s. The proportions of adjusted entries (1%, 2%, 5%, 10%, 20%, 50%, 80%, and 95%) therefore refer to the percentage of characters coded as present that are replaced in this instance. For example, a simulated taxon with 40 of 100 characters coded as present (40 1s, 60 0s) will have 1, 2, 4, 8, 20, 32, and 38 of those presences randomly replaced with absences (1s to 0s) for the closest approximation to the set proportions of adjusted entries. Taxa with fewer present entries will therefore be tested for fewer than eight levels of adjusted entries. Furthermore, in taxa with few characters present, pseudoreplication needs to be avoided; 100 random iterations selecting 1 character out of 40 will cause duplication of search results. At the lowest proportion of introduced absences in taxa with fewer than 100 presences (which will always be one absolute change), iterations were therefore run for each character present rather than 100 random iterations.

**Figure 1.**
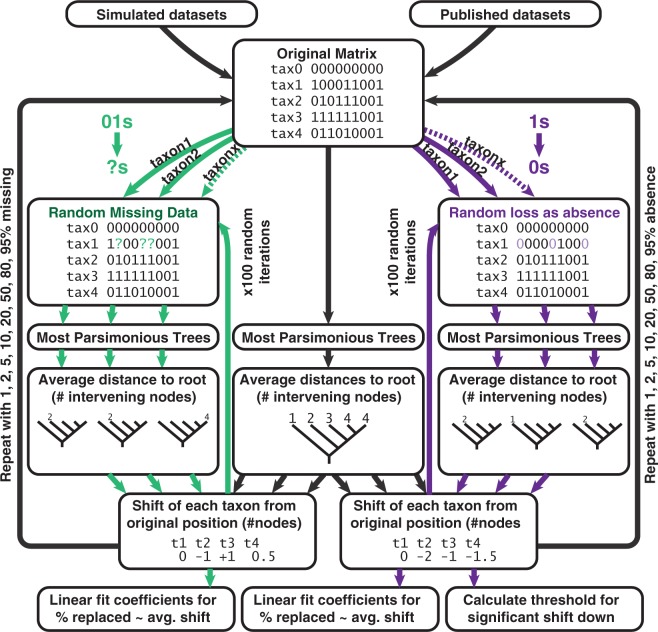
Schematic representation of the stages of the experimental workflow used to investigate the effects of different types of data manipulation (introduced missing data and introduced absences) on the positions of taxa.

Following replacement of entries, new searches are conducted. For each simulated data matrix of 20 taxa, 15,200 searches are therefore necessary for random missing data (19 ingroup taxa with eight levels of missing data and 100 random iterations) and slightly less for introduced absences. Following each of these searches, the position of the manipulated taxon is calculated once again and compared with its original position. The shift of the taxon can then be calculated, both in terms of its magnitude and direction (whether it moves up the tree or down the tree relative to the root and original position, and if so, how far). The stages of the analysis are outlined in Figure 1 and are executed in a script for TNT (Supplementary Script, Dryad http://dx.doi.org/10.5061/dryad.7tq20).

To test whether a taxon has shifted significantly from its original position, two non-parametric tests are applied—a one sample Wilcoxon signed rank test using the magnitudes of the shift of a taxon from its original position for each of 100 random iterations and a binomial test using counts of up or down movement of a taxon from its original position in the 100 random iterations. For random missing data, the null expectations are that there will be no systematic shifts. Introduced absences however are expected to cause taxa to shift down trees toward the root as more absent entries are introduced because they will become more similar to the outgroup condition. Thresholds are established by identifying the lowest level of entries replaced that causes a taxon to shift significantly down the tree. Some taxa, however, cannot move down a tree, for example, any single taxa closest to the root. These, and taxa with two or fewer present characters, are retained in the searches, but excluded from calculation of thresholds.

The test for introduction of absences is not, however, directly comparable to the introduction of random missing data; present characters are preferentially selected for replacement (replacement of 1s with 0s compared with replacement of 0s and 1s with ?s). A further test was therefore applied by selecting only present entries for replacement with missing entries (replacement of 1s with ?s) to directly compare with replacement of 1s with 0s.

The opposite test was used to investigate the effect of interpreting absent characters as missing (0s to ?s). In this case of overcautious coding, the same rationale is used and the same levels of missing data are used, but only absent entries are placed in the pool of entries available for manipulation. In this case and others investigating upward shifts, it is the taxa furthest from the root (i.e., distal) in the original tree that are excluded from the calculation of thresholds up.

### Application to Empirical Data

The introduction of random absences is slightly more complicated for real data sets. Firstly, not all characters are binary. This does not present a problem for the analysis as long as the 0 state defines absence of a character. This relates to the second problem—not all characters are absence–presence characters. Characters for which the 0 state represents some reduction or loss (e.g., 1 appendage rather than 2 or 3) are placed in the pool for the introduction of absences alongside straight absence–presence characters, whereas those that do not (e.g., the color, or position of a structure) are excluded yet they are retained for the searches.

Empirical data sets that contain extinct soft-bodied taxa (i.e., Daley et al. 2009; Wills et al. 2012; Legg et al. 2012; Ma et al. 2013) also offer the possibility of investigating how actual fossil taxa might have been affected by strategies for coding non-preserved characters. If taphonomic loss of data through fossilization causes low placement in phylogenies, the opposite should be true, that is addition of characters to fossil taxa causes higher placement in phylogenies. By replacing absent or missing entries of fossil taxa with present entries (0s or ?s replaced with 1s), it is possible to investigate the direction of taxon shifts and establish thresholds for the number of miscoded entries that result in significant shift from original position. This test required some adjustment for replacement of missing entries with presences. Entries coded as inapplicable (−) are highly unlikely to be coded as such because of non-preservation, rather they are coded as such for logical reasons of character hierarchy. They are therefore treated as “hard missing” and are not included in the pool of missing characters available for replacement with presence. In the case of multistate characters where 0 represents absence, it is necessary to replace 0 with a presence that represents all possible presence entries (e.g., a zero absence is replaced with [12345] for a character with six states). Furthermore, given the size of the combined extinct and extant data set of Legg et al. (2012) and the time taken for each search, drifting methods were used to find trees (Goloboff et al. 2008) with implied weighting (k=3), as utilized by the original authors.

## Results

### Taxon Drift

**Figure 2.**
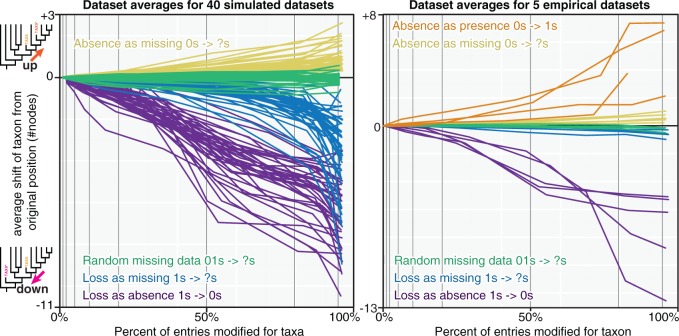
Changing positions of taxa following different strategies of character replacement. Lines represent data set averages. Absences as presence for extinct taxa only.

**Figure 3.**
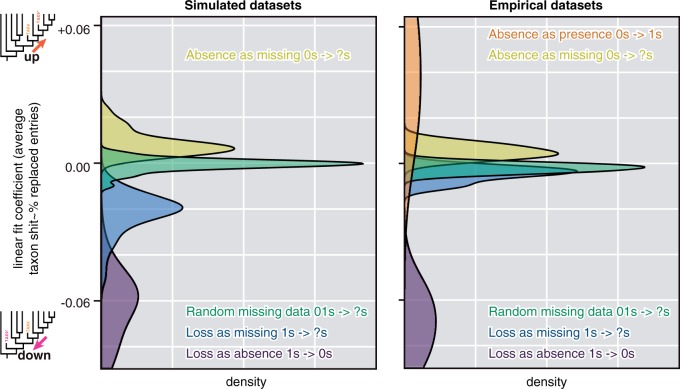
Distribution of linear fit coefficients for percent missing data against average taxon shift for data sets (40 simulated data sets, 5 empirical data sets, and 4 extinct only data sets).

The introduction of random missing data to simulated taxa (replacing 0s and 1s with ?s) fits the expected null hypothesis: individual taxa drift up or down a tree with equal probability, relative to their original position and the root. As more missing data are introduced to a taxon, more noise is introduced and the magnitude of drift from the original position is greater, but these shifts are symmetrical on a gross level (Fig. 2). This balance of drift up and down is statistically supported (linear fit coefficients with zero intercept for percent missing data against average taxon shift for each simulated data set do not differ significantly from zero; one sample t-test P=0.083, n=40; Fig. 3). The same pattern was observed for empirical taxa from published morphological data sets (Fig. 2), which fall well within the range of simulated data (two sample t-test for empirical and simulated coefficients P=0.28; Fig. 3).

The introduction of random absences to simulated taxa (replacing 1s with 0s) also fits the expected null hypothesis: individual taxa shift down the tree toward the root relative to their original position as more present entries are replaced (Fig. 2). This shift is consistently down trees—linear fit coefficients with zero intercepts for percentage entries replaced against average taxon shift are significantly less than zero (one-sample t-test $P=2.2\times 10^{-16}$, n=40; Fig. 3). Empirical data exhibit the same relationship (Fig. 2) and fall within the range of simulated data (two sample t-test for empirical and simulated coefficients P=0.23; Fig. 3).

The corresponding treatment of replacement of present entries with missing data (1s to ?s) also exhibits consistent downward shift of taxa, toward the root (Fig. 2). This shift is also significant (one sample t-test for linear coefficients with zero intercepts, $P=5.8\times 10^{-13}$, n=40), but of a lesser magnitude than that seen for random absences (paired two-sample t-test for coefficients for random absences and selected absences, $P=2.2\times 10^{-16}$; Fig. 3). Empirical data sets do not quite follow the same pattern; whereas linear fit coefficients are significantly below zero (one sample t-test, P=0.031, n=5), they exhibit less downward shift than simulated data sets undergoing the same treatment (two-sample t-test $P=9.9\times 10^{-7}$) and are not significantly different from completely random missing data (paired two-sample t-test for empirical data P=0.05, n=5; Figs. 2 and 3).

Selective replacement of absences with missing data (replacement of 0s with ?s) resulted in drift of simulated taxa up trees, away from the root, relative to their original position (Fig. 2). Linear fit coefficients for the simulated data sets are significantly positive (one-sample t-test $P=5.8\times 10^{-13}$, n=40) and fall significantly outside the range of random missing data (paired two-sample t-test $P=2.2\times 10^{-16}$, n=40; Fig. 3). The same is true of empirical data sets (P=0.0052 and P=0.0072, respectively). They seemingly do not shift up as dramatically as the simulated taxa undergoing the same treatment but this difference is not significant (two-sample t-test P=0.070).

Approaching the problem from the opposite direction using fossil taxa demonstrates that introduction of random presences (replacing 0s with 1s) caused taxa to drift up trees relative to their original position, away from the root (Fig. 2). This shift is significant (linear fit coefficients one sample t-test, P=0.047, n=4) and outside the range of shift seen in random missing data (two-sample t-test, P=0.044, P=0.041 for simulated and empirical data sets, respectively; Fig. 3). Replacing missing entries of fossil taxa with presences (?s to 1s) was only possible for two data sets (Legg et al. 2012; Wills et al. 2012) given the low number of missing entries for the predominately fossil data sets (Daley et al. 2009; Ma et al. 2013). Under these circumstances, one data set showed upward drift of the same magnitude as replacement of absences with presences (Legg et al. 2012) whereas the other showed more balanced drift (Wills et al. 2012).

### Thresholds

**Figure 4.**
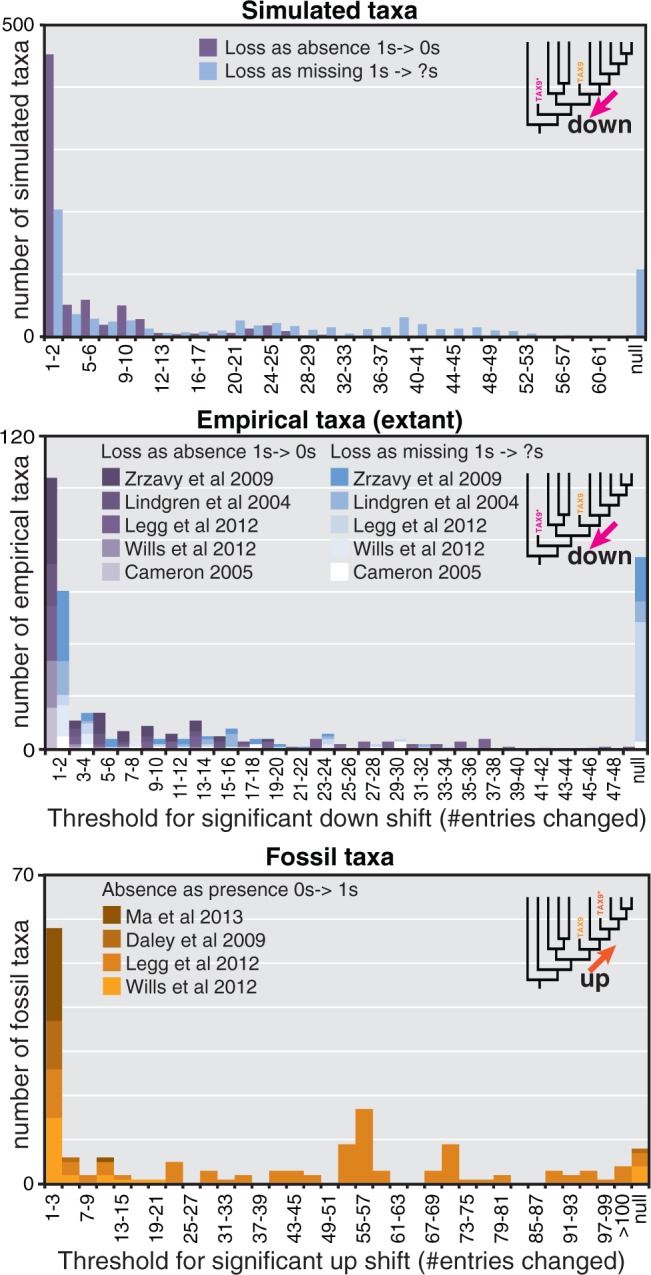
Histogram for distribution of thresholds for significant shift of taxa (absolute number of entries replaced). Null represents those taxa that do not reach a threshold.

As any individual taxon, simulated or empirical, has increasing amounts of absences introduced (replacement of 1s with 0s) and drifts further down a tree (Fig. 2), it should cross a threshold at some point where the shift from its original position becomes significant. For the 760 simulated ingroup taxa from 40 data sets, the distribution of these significant thresholds is very skewed with a long tail—the majority of taxa have a threshold of either 1 or 2 absolute introduced absences, which is less than 6% of present entries (Fig. 4). The same pattern is observed at the rank of data set—29 of 40 have a median threshold of 1 or 2 absolute introduced absences. The same pattern is seen again in the taxa from empirical morphological data sets. The combined 199 taxa from five published data sets have a median threshold of two absolute introduced absences causing significant shift down trees (less than 9% of present entries become absences; Fig. 4). Not all of the empirical data sets are the same however (Table 1). Four of the five fit the general pattern of low thresholds, but the arthropod data set of Legg et al. (2012) has a higher median threshold of absolute entries (13). This most likely reflects the much larger number of present entries in taxa from this matrix (average of 52) compared with taxa from the other empirical data sets (average of 23), although the thresholds for percentage number of entries remain higher (median 21%).

A marked difference is seen in the thresholds for significant downward movement when present entries are replaced with missing data (replacing 1s with ?s). 100% of simulated taxa with introduced absences (1s to 0s) reach a threshold for significant downward shift and have a median of two entries replaced (6% of presences). Only 87% of taxa with introduced missing data (1s to ?s) reach a threshold however, and those have much higher medians: 10 missing entries, 50% of presences (Fig. 4). The pattern of thresholds for introduced missing data is more varied with regards to empirical data; all the extant taxa of Wills et al. (2012) reach a threshold and they have a low median (two entries) whereas only 32% of extant taxa from Legg et al. (2012) reach a threshold. Overall, 127 of 201 empirical extant taxa (63%) reach a threshold for significant movement down when missing data are introduced (1s to ?s), the median of those thresholds being three replacements (14% of present).

In the same sense that thresholds exist for the downward shift of taxa when presences are removed, thresholds should exist for the upward shift of taxa when absences are replaced. When absent entries are replaced with missing entries (0s to ?s), only 422 of 681 simulated taxa (62%) reached a threshold for upward shift, the median of which is six entries replaced (10% of absent entries). Extant empirical taxa followed roughly the same pattern; far fewer reached a threshold for significant shift (44%, 86 of 197), but those that do generally have low threshold values (median of two entries replaced, 8% of absent entries). The data set of Legg et al. (2012) comprises more robust placements with only 18% of taxa reaching significant threshold, and those have a high median (47 entries replaced, 35% of absent entries).

Addition of presences to fossil taxa (replacing 0s with 1s) caused greater shift up trees. 95% of taxa reached a threshold at which they shift significantly up from their original position with a median 22 entries (25% of absent entries). The taxa of Legg et al. (2012) were again comparatively robust (median of 55, 50% of absent entries) but in this instance they represent the majority (61% of all fossil taxa). The fossil taxa from the other three smaller data sets (Daley et al. 2009; Ma et al. 2013; Wills et al. 2012) have appreciably lower thresholds (median of two entries, 11% of absent entries). Replacement of missing entries in fossil taxa with presences (?s to 1s) was only possible for two data sets which had enough missing entries. 50% of fossil taxa from Wills et al. (2012) met the threshold, but the thresholds were low (median of 2.5 entries, 8% of missing entries). 92% of taxa of from Legg et al. (2012) met the threshold, but with relatively higher thresholds (median of 82 entries, 50% of missing entries).

## Discussion

The introduction of random absences to simulated taxa (replacing 1s with 0s) was found to cause taxa to shift down trees, toward the root, as more entries are replaced. This is not surprising in itself and fits the expected null hypothesis. Far more surprising is the extremely low absolute thresholds at which simulated taxa shift significantly down trees—median of two entries replaced, 6% of present entries. Replacement of present entries with missing data in the same fashion (1s to ?s) also causes taxa to drift down trees, but not to the same degree, and the thresholds are much higher. This phenomenon of sensitivity to introduced absences was observed not only in the simulated taxa but also in the extant empirical taxa. 199 empirical taxa from five data sets of soft-bodied clades have a median threshold of two replacements (1s with 0s) causing significant shift of taxa (11% of present entries); thresholds for replacement with missing entries are again much higher. These observed differences between coding strategy indicate that accurately recognizing absences and missing data will have a massive impact on the reconstruction of fossil phylogenies, not only in terms of accuracy but also in terms of systematic biases. The low thresholds for taxon shift mean that only a few misinterpretations are necessary for taxa to be placed significantly lower in trees, closer to the root, than they actually belong. When the problem is approached from the opposite direction, the converse trend is observed: empirical soft-bodied fossil taxa drift up trees as absent entries are replaced with presences; 95% reach thresholds for significant shift up with a median of two replacements (11% of absences) for 56 taxa from small- and medium-sized data sets. Each one of these taxa represents a real-world example where just a few taphonomic losses interpreted as absences will have caused a significant shift in the affinity.

How will this high sensitivity to coding strategy affect evolutionary inferences drawn from fossils? The inevitable loss of morphology during preservation of fossils means that they will always be afflicted by large amounts of missing data. Recognizing non-preserved anatomical synapomorphies as missing rather than absent, or vice versa, is generally problematic for fossils. It is especially problematic for soft-bodied fossils, when we often have no *a priori* reason to believe that some features will be preserved when others will not. Nevertheless, from the analyses presented here, it is apparent that only two miscoded entries (i.e., two non-preserved characters interpreted as absent) are necessary for a taxon to be placed significantly lower in a tree that it actually belongs, that is to say, closer to the root. This is true for the simulated taxa, and also identified for real-world examples of empirical taxa, both extant sliding down trees and extinct sliding back up. Such positioning will have massive ramifications for our understanding of the phylogenetic placements of fossils and the evolutionary events that rely on those placements. After all, it is the very position of an extinct taxon in a phylogenetic framework relative to extant taxa and the root that reveals its evolutionary informativeness, be that through calibration of molecular clocks or reconstructing morphological transitions. A fossil taxon shifting down a few nodes will distort inferences in both these instances (Sansom and Wills 2013).

This phenomenon of stem-ward slippage was first observed in early chordates where systematic decay biases cause loss of synapomorphic characters during decay leaving plesiomorphic remains that can be interpreted as artifactually primitive when fossilized (Sansom et al. 2010a, 2011). In this instance, an unexpected correlation between synapomorphic rank of characters and decay susceptibility of characters causes bias in the interpretation of fossil affinity. It is demonstrated here, however, that even when no such decay bias exists, simple random misinterpretation of characters, even just two, will also cause significant stem-ward slippage.

Unless taken into account, the phenomenon of stem-ward slippage risks undermining our ability to use fossils in evolutionary contexts (Sansom and Wills 2013), especially soft-bodied forms. What can be done to ensure secure and sensible fossil placements? One possibility is a more cautious approach to treatment of absences, that is, when in doubt, code a non-preserved character as ? rather than 0. The results of analyses presented here, however, indicate that over-cautious coding causes the opposite problem. As more absent entries are interpreted as missing (0s replaced with ?s), taxa drift up trees, away from their root, relative to their original position. Although fewer taxa reach thresholds for significant shift (62% and 44% of simulated and empirical taxa, respectively), thresholds are low—median of six entries, 10% of absent entries for simulated taxa, and median of two entries, 8% of absent entries for empirical taxa. One could hope that the downward shift caused by misinterpreted missing entries would be balanced by the upward shift of misinterpreted absent entries, but the effects are not of the same magnitude, nor would they be expected to be taxonomically consistent. Instead of closing one's eyes and hoping for the best, a more sensible strategy is interpretation of non-preserved characters in light of taphonomic frameworks. Each absent and missing entry of a soft-bodied taxon requires explicit justification, just as is the case for presence entries. This is true for both formal phylogenetic investigation (e.g., Daley et al. 2009; Legg et al. 2012; Wills et al. 2012; Ma et al. 2013) and less constrained taxonomic assignment (e.g., Smith and Caron 2010; Conway Morris and Caron 2012; Caron et al. 2013). Indeed this is likely to be even more important for informal analyses where there are often far fewer characters available and any single error will represent a far higher proportion of characters present, and its effects more dramatic.

Justification for interpreted absences can be achieved in a number of ways. Reference to actualistic character decay profiles, for example the ‘Atlas of vertebrate decay’ (Sansom et al. 2013), enables recognition of partially decayed characters and the relative likelihood of character preservation (Sansom et al. 2010b). Furthermore, relative character decay profiles can be used to distinguish characters that are likely to be missing or absent using the concept of taphonomic coherency (Sansom et al. 2010a, 2011; Sansom 2014). For example, the absence of a cartilaginous cranium in the fossil vertebrate *Myllokunmingia* is likely to be a real absence because other cartilaginous characters which are more likely to be lost to decay (i.e., branchial cartilages) are preserved, thus indicating that if a cranium was present, we should expect to see it (Sansom et al. 2011; Sansom 2014). Similarly, the fossil lamprey *Mayomyzon* does not yield a full complement of lamprey characters indicating it could be a stem-petromyzontid; when compared with lamprey character decay profiles however, the missing lamprey characters are all lost early (Sansom et al. 2011). These two fossils are therefore best interpreted as stem-vertebrates and total-group petromyzontids, respectively. An alternative form of taphonomic framework is the concept of taphonomic thresholds whereby preservation of particular key taxa at identified stages of decay can be used as an indication of the taphonomic fidelity of a particular deposit (Briggs and Kear 1993). For example, preservation of polychaetes comparable to Stage 2 decay in actualistic experiments (Briggs and Kear 1993) would argue for a higher fidelity of preservation of other taxa belonging to the same layer. All of these suggested solutions have the same underlying rationale—use of rigorous and explicit taphonomic frameworks to inform decisions regarding the non-preserved characters in soft-bodied fossils. This will make it possible to eliminate the possibility of non-preservation being conflated with absence. Only following those steps will it be possible to place confidence in the phylogenetic affinity of soft-bodied fossils and the evolutionary conclusions drawn from them.

## Conclusions

It is well understood that absence of evidence does not necessarily equate to evidence of absence (Walton 1992). Absence is inherent and ubiquitous in paleontological data due to non-preservation, yet its treatment is rarely discussed explicitly. It has been demonstrated through the analyses presented here that the reconstruction of the phylogenetic affinity of fossil taxa is extremely sensitive to the strategy used to interpret non-preserved characters. Whether treated as missing or absent, taphonomic loss of present characters causes taxa to slide down trees, toward the root. In the case of non-preservation interpreted as absence, a median of two missing characters interpreted as absent causes significantly low phylogenetic placement of taxa, both simulated and extant. Such a small threshold of errors is easily conceivable in a fossil record rife with non-preserved characters and thus non-preservation could be systematically distorting the evolutionary inferences we draw from soft-bodied fossils. Indeed the same but opposite thresholds are found for empirical soft-bodied fossil taxa drifting up trees when absences are retrospectively reinterpreted as presences. To make the best use of the unique data that the exceptionally preserved soft tissue fossil record yields, it is recommended that interpretation of non-preserved characters as absent is done explicitly and where possible with reference to taphonomic frameworks. Only then can we have confidence that our interpretation of any particular fossil as an informative stem-group representative or the first representative of a group is secure, and not a merely artifact of data loss through fossilization.

## Supplementary Material

Data available from the Dryad Digital Repository: http://dx.doi.org/10.5061/dryad.7tq20.

## Funding

This work was supported by the Natural Environment Research Council through a fellowship [NE/I020253/1].

## References

[B1] Bartels C., Briggs D.E.G., Brassel G. (2009). The fossils of the Hunsrück Slate: marine life in the Devonian.

[B2] Beland P., Arsenault M. (1985). Scaumenelisation de l'Acanthodii *Triazeugacanthus affinis* (Whiteaves) de la Formation d'Escumina (Devonien Superieur de Miguasha, Quebec): revision do *Scaumenella mesacanthi* Graham-Smith. Can. J. Earth Sci..

[B3] Brazeau M.D. (2011). Problematic character coding methods in morphology and their effects. Biol. J. Linn. Soc..

[B4] Briggs D.E.G., Erwin D.H., Collier F.J. (1994). The fossils of the Burgess Shale.

[B5] Briggs D.E.G., Kear A.J. (1993). Decay and preservation of polychaetes: taphonomic thresholds in soft-bodied organisms. Paleobiology.

[B6] Cameron C.B. (2005). A phylogeny of the hemichordates based on morphological characters. Can. J. Zool..

[B7] Caron J.-B., Conway Morris S., Cameron C.B. (2013). Tubicolous enteropneusts from the Cambrian period. Nature.

[B8] Conway Morris S., Caron J.-B. (2012). *Pikaia gracilens* Walcott, a stem-group chordate from the Middle Cambrian of British Columbia. Biol. Rev..

[B9] Daley A.C., Budd G.E., Caron J.-B., Edgecombe G.D., Collins D. (2009). The Burgess Shale anomalocaridid *Hurdia* and its significance for early euarthropod evolution. Science.

[B10] Donoghue P.C.J., Benton M.J. (2007). Rocks and clocks: calibrating the Tree of Life using fossils and molecules. Trends Ecol. Evol..

[B11] Donoghue P.C.J., Purnell M.A. (2009). Distinguishing heat from light in the debate over controversial fossils. BioEssays.

[B12] Forey P., Kitching I., Scotland R.W., Pennington R.T. (2000). Experiments in coding multistate characters. Homology and systematics: coding characters for phylogenetic analysis.

[B13] Goloboff P.A., Farris J.S., Nixon K.C. (2008). TNT, a free program for phylogenetic analysis. Cladistics.

[B14] Hou X., Siveter D.J., Williams M., Walossek D., Bergström J. (1996). Appendages of the arthropod *Kunmingella* from the Early Cambrian of China: its bearing on the systematic position of the Bradoriida and the fossil record of the Ostracoda. Phil. Trans. R. Soc. B.

[B15] Hou X., Aldridge R.J., Bergström J., Siveter D.J., Siveter D.J., Feng X. (2004). The Cambrian fossils of Chengjian, China: the flowering of early animal life.

[B16] Hou X., Williams M., Siveter D.J., Siveter D.J., Aldridge R.J., Sansom R.S. (2010). Soft-part anatomy of the Early Cambrian bivalved arthropods *Kunyangella* and *Kunmingella*: significance for the phylogenetic relationships of the Bradoriida. Proc. R. Soc. B.

[B17] Huelsenbeck J. (1991). When are fossils better than extant taxa in phylogenetic analysis?. Syst. Biol..

[B18] Kearney M., Clark J.M. (2003). Problems due to missing data in phylogenetic analyses including fossils: a critical review. J. Vertebr. Paleontol..

[B19] Legg D.A., Sutton M.D., Edgecombe G.D., Caron J.-B. (2012). Cambrian bivalved arthropod reveals origin of arthrodization. Proc. R. Soc. B.

[B20] Lindgren A.R., Giribet G., Nishiguchi M.K. (2004). A combined approach to the phylogeny of Cephalopoda (Mollusca). Cladistics.

[B21] Ma X., Edgecombe D., Legg D.A., Hou X. (2013). The morphology and phylogenetic position of the Cambrian lobopodian *Diania cactiformis*. J. Syst. Palaeontol..

[B22] Murdoch D.J.E., Donoghue P.C.J. (2011). Evolutionary origins of animal skeletal biomineralization. Cells Tissues Organs.

[B23] Paul C.R.C., Donovan S.K., Paul C.R.C. (1998). Adequacy, completeness and the fossil record. The adequacy of the fossil record.

[B24] Prevosti F.J., Chemisquy M.A. (2010). The impact of missing data on real morphological phylogenies: influence of the number and distribution of missing entries. Cladistics.

[B25] Sansom R.S., Laflamme M., Schiffbauer J.D., Darroch S.A.F. (2014). Experimental decay of soft tissues. Reading and writing the fossil record: preservational pathways to exceptional fossilization.

[B26] Sansom R.S., Wills M.A. (2013). Fossilization causes organisms to appear erroneously primitive by distorting evolutionary trees. Sci. Rep..

[B27] Sansom R.S., Gabbott S.E., Purnell M.A. (2010a). Non-random decay of chordate characters causes bias in fossil interpretation. Nature.

[B28] Sansom R.S., Gabbott S.E., Purnell M.A. (2011). Decay of vertebrate characters in hagfish and lamprey (Cyclostomata) and the implications for the vertebrate fossil record. Proc. R. Soc. B.

[B29] Sansom R.S., Gabbott S.E., Purnell M.A. (2013). Atlas of vertebrate decay: a visual and taphonomic guide to fossil interpretation. Palaeontology.

[B30] Sansom R.S., Freedman K., Gabbott S.E., Aldridge R.J., Purnell M.A. (2010b). Taphonomy and affinity of an enigmatic Silurian vertebrate, *Jamoytius kerwoodi* White. Palaeontology.

[B31] Scotland R.W., Olmstead R.G., Bennett J.R. (2003). Phylogeny reconstruction: The role of morphology. Systematic Biology.

[B32] Shabica C.W., Hay A.A. (1997). Richardson's guide to the fossil fauna of Mazon Creek.

[B33] Shu D., Vannier J.M.C., Luo H., Chen L., Zhang X., Hu S. (1999). Anatomy and lifestyle of *Kunmingella* (Arthropoda, Bradoriida) from the Chengjiang fossil Lagerstätte (Loer Cambrian, southwest China). Lethaia.

[B34] Strong E.E., Lipscomb D. (1999). Character coding and inapplicable data. Cladistics.

[B35] Smith M.R., Caron J.-B. (2010). Primitive soft-bodied cephalopods from the Cambrian. Nature.

[B36] Walton D. (1992). Nonfallacious arguments from ignorance. Am. Phil. Quart..

[B37] Wiens J.J. (1998). Does adding characters with missing data increase or decrease phylogenetic accuracy?. Syst. Biol..

[B38] Wiens J.J. (2003a). Missing data, incomplete taxa, and phylogenetic accuracy. Syst. Biol..

[B39] Wiens J.J. (2003b). Incomplete taxa, incomplete characters, and phylogenetic accuracy: is there a missing data problem?. J. Vertebr. Paleontol..

[B40] Wiens J.J. (2004). The role of morphological data in phylogeny reconstruction. Systematic Biology.

[B41] Wiens J.J. (2006). Missing data and the design of phylogenetic analyses. J. Biomed. Inform..

[B42] Wiens J.J., Morrill M.C. (2011). Missing data in phylogenetic analysis: reconciling results from simulations and empirical data. Syst. Biol..

[B43] Wills M.A., Gerber S., Ruta M., Hughes M. (2012). The disparity of priapulid, archaeopriapulid and palaeoscolecid worms in light of new data. J. Evol. Biol..

[B44] Zhou Z., Barrett P.M., Hilton J. (2003). An exceptionally preserved Lower Cretaceous Ecosystem. Nature.

[B45] Zrzavý J., Riìha P., Piaìlek L., Janouškovec J. (2009). Phylogeny of Annelida (Lophotrochozoa): total-evidence analysis of morphology and genes. BMC Evol. Biol..

